# Factors Driving the American Aesthetic Tourism to South of the Border

**Published:** 2019-01

**Authors:** Onesimo CUAMEA, Karen RAMOS

**Affiliations:** 1. Facultad de Turismo y Mercadotecnia, Universidad Autonoma de Baja California, Tijuana, Mexico; 2. Facultad de Contaduria y Administracion. Universidad Autonoma de Baja California, Tijuana, Mexico

**Keywords:** Aesthetic tourism, Predictors, Border region, International tourism

## Abstract

**Background:**

The problem consists in the lack of knowledge of the factors that tourists choose the clinics of a foreign city for their aesthetics treatments, especially at a frontier where a developed and developing country coexist and interact.

**Methods:**

The information was obtained on 2016, by applying an exit pool survey to a selected sample of 385 visitors- patients from clinics in the Tijuana, Mexico. Sixteen items were included in the exploratory factor analysis.

**Results:**

Four key factors influenced the decision: Quality & Prestige of Clinic and Surgeon, Integration with Tourism Sector, Destination Image and Border Interaction. All the elements had a load factor greater than 0.56 which proved the actual fit in the factor analysis and none of the included variables is trivial.

**Conclusion:**

Allow maintaining or increasing the attractiveness of a border city for aesthetic tourism, the administrators of the clinics establish cooperation agreements with entrepreneurs of the tourism sector to benefit their patients.

## Introduction

All foreigners visiting a country for health reasons are considered tourists and contribute to the accounting of spending generated by international tourism (1). It is necessary to specify the different categories in which travelers can be classified for medical or health reasons. The “tourist” is the person who temporarily moves out of their place of residence (habitual environment) and stays in the place visited more than one day performing at least one overnight at the destination. However, if the travel is done on the fringes or border areas of another country and returns to their place of habitual residence without having spent the night, then it is considered “frontier excursionist” or “international excursionist” (1).

The most popular destinations for cosmetic procedures are India and Mexico, and the main reason for traveling to these countries is the low price (2). Americans prefer to visit Mexico, Costa Rica or Panama for dental or cosmetic treatments. In addition to geographic proximity, Mexico offers between 25% and 35% discount on these procedures (2). The main incentives for traveling to the south of the Mexican diaspora in America are the high costs of medical care, barriers to care for minorities, low income, and familiarity with the Mexican health system (3).

Like any emerging market niche, aesthetic tourism should be further studied and especially at the local level and characterizing the demand, based on factors such as age, gender, place of residence, medical treatment, medical coverage plans available and type and trip configuration, among others (4).

The Pollard’s conceptual model of attractiveness of destinations for medical tourism (Table 1), establishes seven dimensions that include 22 factors to measure it (5).

**Table 1:** Model of destination attractiveness

***Dimensions***	***Factors***
Geographical proximity	Travel time, ease of airport Access, barriers to entry
Cultural proximity	Language, food, religion, customs and practice
Destination image	Place myths
Destination infrastructure	Accommodation, internal travel, support services
Destination environment	Tourism attractions, facilities, climate
Risk and reward	Safety, guarantee, track records, outcome
Price	Cost of stay, cost of treatment, cost of travel, insurance

Source: Pollard, (2012)

This model excludes the factors of high quality, technology and low cost because they are repeatedly used by all companies and countries to attract them. In an immature and unsophisticated medical tourism market, the destinations (or health care providers) that will succeed are those who understand and develop the concept of “destination attractiveness” (5). From the lack of knowledge about factors by which tourists from a border region choose the clinics of a foreign city for their aesthetic treatments, to achieve the following objectives. This study aimed to identify the socioeconomic and sociodemographic profile of aesthetic tourism that visits Tijuana; and Hierarchize the factors that visitors-patients take into account to choose a clinic for some procedure or aesthetic treatment in the city of Tijuana, Mexico.

## Methods

Based on Pollard’s model (5) and considering the border area of study, it was decided to include the same seven dimensions but reducing it from 22 to 16 aspects, eliminating those that do not apply in the geographic context and the regulatory framework (Table 2), then, using the quantitative method and applying the survey technique to carry out an exploratory factor analysis.

**Table 2:** Selected factors that visitors take into account to choose a clinic

	***Dimensions***	***Factors***
1	Geographical proximity	The location of the plastic surgery clinic in the city
2	Cultural proximity	The recommendation of a friend or relative
		The care offered by the plastic surgery clinic
		Agreement with restaurants for their patients
		Staff speaks English language
3	Destination image	Reputation of the local police
		Perception of the urban image of the city
		Urban signs, traffic volume and street conditions
4	Destination infrastructure	The hospital facilities of the plastics surgery clinic
		Provide transportation to their patients
5	Destination environment	Agreement with hotels to host their patients
		Provide tourist information to its patients
		Agreement with a Spa for patients
6	Risk and reward	The confidence generated by the surgeon
		The prestige of the plastic surgery clinic
7	Price	The Price of treatment in the plastic surgery clinic

Source: Own elaboration based on Pollard, (2012)

### Sampling Unit

It was determined to survey patients at the exit of aesthetics clinics. Only were included tourists or excursionist residing in Mexico and abroad who accepted to respond to the survey.

### Definition of Sample Size and Procedure for Data Collection

To define the sample size a confidence level of 95% and a margin of error of ± 5% were established, resulting in 380 patients being surveyed. All the participants gave their consent to be a part of the study. In order to validate and design the final instrument, three pilot surveys were carried out in the months of Mar and Apr of 2016, each pilot test was applied with a sample of 40 visitors.

The final survey includes the socioeconomic and sociodemographic data and sixteen factors to choose an aesthetic clinic; a five-point Likert scale was used with the following answers: 1 = Not important, 2 = Less important, 3 = Important, 4 =Very Important and 5= Extremely Important.

### Reliability of Instrument

The results of the reliability analysis -Cronbach’s Alpha Value test demonstrates the consistency between the measurements scales used in the sixteen variables used in the research. A score of 1.0 on the Cronbach Alpha indicates 100 percent reliability. The result obtained from 0.726 is above the generally accepted score of Nunnally (6) of 0.7; which shows the reliability of the questionnaire.

## Results

The sociodemographic profile of the respondents (Table 3) showed that, overall, 66.74% are between 20 and 40 yr old. 95.6% of the respondents were female.

**Table 3:** Sociodemographic characteristics of participants

***Variable***	***Characteristics***	***Frequency***	***%***
Age(yr)	20 to 30	119	30.90
	31 to 40	138	35.84
	41 to 50	81	21.03
	51 to 60	38	9.87
	61 and above	9	2.33
Gender	Male	17	4.40
	Female	368	95.60
Type of visitor	Tourist	108	28.10
	Excursionist	277	71.90
Ethnic profile	Hispanic emigrated to the US	186	48.30
	Hispanic born in the US	97	25.30
	Asian	1	0.30
	Caucasian	38	9.90
	African-American	3	0.80
	Mexican	60	15.60
Method of payment	Cash	334	86.80
	Credit or debit card	50	13.00
	Medical insurance	1	0.30
Occupation	Self-employed	31	8.10
	Employee	227	53.00
	Student	23	6.00
	Home	98	25.50
	Retired	6	0.50
Place of residence	California, US	322	83.60
	Other US states	6	1.50
	Baja California, Mexico	43	11.20
	Other Mexico States	14	3.63
Monthly income (US dollars)	$ 800 dollars and below	19	4.93
	$ 801 to $ 1600	50	12.98
	$ 1601 to $ 2400	57	14.80
	$ 2401 to $ 3200	80	20.77
	$ 3201 and above	111	28.83
	Did not declare income	68	17.66

Only 28.1% correspond to tourists (people who spend at least one night in the city) and 71.9% are day visitors or excursionist. The visitor’s ethnic profile is represented mainly by Hispanics with 73.6.7%, either Hispanics who immigrated to the United States, or children of Hispanics who emigrated but who were born in the United States. Cash was the principal method of payment.

The majority was employees, residents of Southern California and the 49.6% reported a monthly income between $ 2401 and $ 3,201 and above.

### Exploratory Factor Analysis

In order to identify the aspects that visitors take into account when choosing an aesthetic clinic, as well as to examine the appropriateness of the data to carry out the exploratory factor analysis, the KMO and Bartlett’s sphericity tests were performed.

If the total result exceeds 0.50 means that factor analysis is useful with the given data (7). The data are adequate for factor analysis due to the value of 0.796 and confirms that a factor analysis is appropriate. Additionally, the level of significance has a very small value (Sig. =0.001) indicating that the variables are highly correlated.

With the purpose of determining the minimum number of factors that account for the maximum variance of the data, the principal component analysis was applied, as shown in Table 4, after reducing the 16 variables and considering only initial eigenvalues greater than one, four representative uncorrelated components together explain 60.33% of the total variance over the decision. The rest of the components with initial eigenvalues smaller than one were discarded because together they explain only 39.67% of cumulative variance.

**Table 4:** Total Variance Explained

***Component***	***Initial Eigenvalues***			***Extraction Sums of Squared Loadings***			***Rotation Sums of Squared Loadings***		
	***Total***	***% of variance***	***Cumulative %***	***Total***	***% of variance***	***Cumulative %***	***Total***	***% of variance***	***Cumulative%***
1	4.219	26.370	26.370	4.219	26.370	26.370	3.873	24.205	24.205
2	2.953	18.457	44.828	2.953	18.457	44.828	2.889	18.058	42.263
3	1.439	8.996	53.824	1.439	8.996	53.824	1.551	9.694	51.957
4	1.042	6.510	60.334	1.042	6.510	60.334	1.340	8.377	60.334
5	.957	5.983	66.317						
6	.859	5.368	71.685						
7	.679	4.242	75.927						
8	.657	4.107	80.034						
9	.590	3.690	83.724						
10	.583	3.645	87.369						
11	.552	3.450	90.819						
12	.454	2.840	93.660						
13	.411	2.568	96.227						
14	.322	2.015	98.243						
15	.210	1.314	99.557						
16	.071	.443	100.000						

Extraction Method: Principal Component Analysis

The idea of rotation is to reduce the number of factors on which the variables under investigation have high loadings. The result of the factor analysis shows four essential components to choose an aesthetical clinic. The rotated component matrix allows identifying the variables that present significant loads in the same factor, enabling the definition of common ones (Table 5).

**Table 5:** Rotated Component Matrix ^a^

***Number***		***Variables***	***Component***			
			***1***	***2***	***3***	***4***
	1	The recommendation of a friend or relative	.945			
	2	The confidence generated by the surgeon	.892			
	3	The care offered by the plastic surgery clinic	.872			
	4	The hospital facilities of the plastic surgery clinic	.819			
	5	The prestige of the plastic surgery clinic	.575			
	6	The price of treatment in the plastic surgery clinic	.565			
	7	Agreement with restaurants for their patients		.804		
	8	Agreement with hotels to host their patients		.797		
	9	Provides tourist information to its patients		.726		
	10	Agreement with a Spa for patients		.710		
	11	Local transportation service to their patients		.678		
	12	Reputation of the local police			.766	
	13	Perception of the urban image of the city			.730	
	14	Urban signs, traffic volume and street conditions			.588	
	15	Staff speak English language				.823
	16	The location of the plastic surgery clinic in the city				.590

Extraction Method: Principal Component analysis.

Rotation Method: Varimax with Kaiser Normalization.

a. Rotation converged in 5 iterations

The first component of relevance, which was called “Quality & Prestige of Clinic and Surgeon Factor”, includes in order of importance and according to their respective factor loading: the recommendation of a friend or relative (.945), the confidence generated by the surgeon (.892), the care offered by the plastic surgery clinic (.872), the hospital facilities of the plastic surgery clinic (.819), the prestige of the plastic surgery clinic (.575) and the price of treatment in the plastic surgery clinic (.565). By itself, this factor accounts for a variance of 26.37%.

The second component determined as “Integration with Tourism Sector Factor” includes aspects such as agreement with restaurants for their patients (.804), agreement with hotels to host their patients (.797), provides tourist information to its patients (.726), agreement with Spas for patients (.710), local transportation service to their patients (.678), and it represents 18.45 % of the variance. Thirdly, appears the component called “Destination Image Factor”, which includes aspects directly related to the reputation of the local police (.766), perception of the urban image of the city (.730) and urban signs, traffic volume and street conditions (.588) explaining the 8.99 % of the variance.

The fourth and final component called “Border Interaction Factor” refers to the fact that the staff speaks English language (.823) and the location of the plastic surgery clinic in the city (.590), explicating the 6.51% of the variance.

## Discussion

The most used factor to attract medical tourism from the supply side is the reduction of medical costs and waiting times, quality of service and international accreditation of medical facilities. While the less used include the reputation of doctors and the social and cultural familiarity of tourists with destinations. With regard to the most used push motivators, on the demand side, are recommendations (from friends, doctors or family) inadequate insurance coverage and the privacy and confidentiality of treatments (8).

The image of tourist destination creates a positive influence on perceived quality and satisfaction since that image influences the expectations that individuals have before travel. When medical tourists perceived low cost and high quality of medical staff, they are likely to be more satisfied with both services provided and the chosen destination (9).

Patients prefer to be treated outside their country when they are more familiar with the foreign language, culture or healthcare system or when services are similar to those offered in their country of residence; this is the typical case of citizens living in border regions. In addition, migrants often prefer to be treated in their home countries (10).

Regarding destination image (i.e. safety and stability), the internet, word-of-mouth, family and friends play an important role by providing recommendations and experiences. In some, the cost of medical treatments, insurance coverage, physicians’ expertise, quality of care and facilities, accreditations, as well as, follow up care are among the most important factors affecting decisions made by medical tourists (11).

The patients tend to make judgments based on the Functional Quality of the service, that is, their experience with people and systems in the healthcare environment associated with performing the procedure. Patients value highly attention, compassion and communication (12). The main factors that influenced the choice of a surgeon were the quality of the preoperative information, the doctor-patient relationship and the results observed by the family or friends (13).

Shorter waiting times, cheaper costs, higher quality healthcare services, other surgeons better understand their wishes are the main reasons for travel abroad for cosmetic surgeries (14). In the case of the European Union, tourists travel to other countries in the same region, mainly because they prefer to be treated near their place of residence, knowledge of the language, the availability, familiarity and accessibility of the required treatment and perceived quality of health care system (15).

In South Africa, the most important factors to choose a destination were quality of service, facilities and accreditation. With lesser importance were placed the quality of accommodation, recommendation of a local doctor and general tourism supply such as tourist attractions and quality of infrastructure (16). Other factors are the cost, reputation of the surgeon, the hospital’s reputation and facilities for recovery (17). In studying the attractiveness of a country as a destination for health tourism, medical tourists favor more the quality of service and the cost of the procedure than the competitiveness and the tourist attitude in the destination (18).

There are four main categories that explain the changes in the travel pattern of this type of tourists: a) the lack of services in the country of origin; b) The cost of treatment in relation to the quality perceived by the user; c) Cultural aspects such as communicating in the same language or the doctor-patient relationship; d) Regional integration that facilitates the movement of people from one country to another (19).

The three main factors taken into account before deciding whether or not to take a trip abroad are “competent physicians”, “high-quality medical care” and “immediate medical treatment when necessary”. The results will be useful for companies directly or indirectly involved with this industry, such as insurance companies, travel agencies, hotels, food and beverage business and medical companies (20).

When analyzing the place of residence of the majority of medical tourists in Tijuana, the main sending cities are Los Angeles, San Diego, Chula Vista, Mexicali and Ensenada (21). The farthest city is located 135 miles and 2 h by car, the costs of a road trip are lower than air travel. Also, the ethnic profile of medical tourist is mainly represented by Hispanics, either born or legally immigrated to the USA.

Due to the Geographical proximity and that there are no flights between those cities, it was determined to exclude the factors of travel time and ease of airport access. In the same way, Secretariat of Foreign Affairs of Mexico (22), states that citizens and legal residents of the United States do not require a visa to enter Mexico (including border areas), which allowed eliminating the barriers to entry factor.

In addition, the Secretariat of Foreign Affairs of Mexico (22), states that citizens and legal residents of the United States do not require a visa to enter Mexico (including border areas). In the same sense, the Mexican Federal Law to Prevent and Eliminate Discrimination states that it is considered discriminatory to deny or condition health care services or to prevent the participation of the patient in decisions about medical or therapeutic treatment (23). In sume, the Secretariat of Tourism of Baja California (24). affirms that 78% arrives in car and just the 25% remains overnight in the city. The lack of insurance portability, which difficult to track medical records, remains a major barrier to the growth of aesthetic tourism (25).

With respect to the Cultural Dimension, and taking into account that, the majority of patient-visitors are of Hispanic origin, for whom the taste, consistency and presentation of Mexican food is familiar and does not generate the sensation of distribution (21, 24), this factor was not included. Religion factor was also removed, because that the Mexican Federal Law to Prevent and Eliminate Discrimination states that it is considered discriminatory to deny or condition health care services or to prevent the participation of the patient in decisions about medical or therapeutic treatment (23).

Regarding the Price and Risk and Reward Dimension, the cost of travel and cost of stay factors were excluded, because the costs of a road trip are lower than air travel. The Secretariat of Tourism of Baja California (24) affirms that 78% arrives in car and just the 25% remains overnight in the city. In this dimension, insurance factor was left out because the aesthetic procedures are not covered by insurance companies, most of the cosmetic surgery services are paid by patients who either have no insurance coverage or whose insurance does not cover this procedure (6).

After carrying out the exploratory factor analysis, the results in this study are in agreement with the findings of different researchers, especially in the first factor called Quality and Prestige of Clinics and Surgeon, which refers to the internal aspects of clinics such as recommendation (11), quality of service (8) (9), high quality of care (15, 20) doctor-patient relationship (13, 16) and facilities, prestige of the surgeon and low cost of the treatment ( 11, 12, 15, 17, 19).

Regarding the third Factor named Destination Image, there are a few studies that incorporate these variables into the literature of aesthetic tourism. Nevertheless, the security and stability in the destination (11), the place myths (5) such as the reputation of the local authorities and the urban infrastructure of the city are taken in to account for aesthetic tourist to choose a destination to do the cosmetic procedure.

The fourth factor titled Border Interaction, due to the characteristics of the border regions; it is integrated by the familiarity and accessibility of the destination and clinics, cultural aspects such as communicating in the same language with the doctor and staff (19), in other words, the knowledge of the tourist language (20). Similar conclusions were found in the studies of the case in the European Union border region (14, 10).

The second factor designated as integration with Tourism Sector, which includes actions that the administrators of cosmetic surgery clinics perform to facilitate the stay and mobility of their patients in the destination, and which resulted from the Model of Attractiveness adaptation (5), specifically the Destination Infrastructure and Destination Environment Dimensions.

These are aspects generally incorporated in previous studies, and considering the importance of these to choose a clinic in this border region, it is advisable to include them in future studies on aesthetic tourism in other regions with similar characteristics.

The factors in the dimension need to be analyzed before applying Pollard′s model in other destinations. The factors left out could be necessary depending on the context and regulatory framework of the city, region or country. If a tourist profile is not defined and connectivity of a destination it’s mainly by air, travel time and airport access shall be included, as well, as the cost of stay and cost of travel factors. On the other hand, according to Mexican Laws religion is not an important factor for medical procedures (23), in different countries could be a preponderant social aspect directly related to the decision to undergo medical procedures,

## Conclusion

The religion factor should be evaluated and more cultural proximity factors would be needed to assess the interaction with medical or aesthetic tourism and the importance of this factor within the decision to choose a clinic in another destination. An evaluation of the whole model is recommended, mainly when the esthetic tourists travel from a developed country to a developing one.

## Ethical considerations

Ethical issues (Including plagiarism, informed consent, misconduct, data fabrication and/or falsification, double publication and/or submission, redundancy, etc.) have been completely observed by the authors.
